# First Report of Gallbladder Volvulus Managed with a Robotic Approach

**DOI:** 10.1155/2019/2189890

**Published:** 2019-07-14

**Authors:** Roberto Bustos, Hassan Mashbari, Antonio Gangemi

**Affiliations:** Division of General, Minimally Invasive and Robotic Surgery, Department of Surgery, University of Illinois at Chicago, 840 S. Wood Street, Suite 435E (MC 958), Chicago, IL 60612, USA

## Abstract

Gallbladder volvulus (GV) is an axial twisting of the gallbladder (GB). If not treated on time, this condition has a mortality up to 6%. It is usually diagnosed intraoperatively, because it can mimic a typical acute cholecystitis. An 81-year-old female patient presented with an acute onset of right upper quadrant pain accompanied with nausea. The patient was admitted to receive treatment for acute cholecystitis after the findings of ultrasound imaging. Robotic-assisted cholecystectomy was the approach chosen. GV was diagnosed after initial diagnostic laparoscopy. Cholecystectomy was performed uneventfully. Indocyanine green fluorescence was used to assess the biliary anatomy. The postoperatory course went uneventful. The patient was discharged home on postoperatory day 2.

## 1. Background

Management of acute cholecystitis has been proven to be safe in the minimally invasive setting, even for elderly patients [1]. Nevertheless, when decision is made to take the patient to the operating room, gallbladder volvulus (GV) is an unexpected finding that usually is discovered intraoperatively [2].

GV is defined as an axial twisting of the gallbladder (GB), with subsequent interruption of the vascular and biliary flow [3]. As a consequence, GB necrosis and inflammation are manifested as an acute abdomen [2]. This condition was described for the first time by Wendell in 1898. Since then, approximately 400 cases have been reported in literature [4]. Throughout the years, an increase of reports has been observed, especially in Japanese literature [3], possibly due to increased life expectancy and the fact that GV is more frequent in the elderly [4].

Robotic-assisted surgery has had an exponential growth in the last two decades, but its use in the acute setting has still to be fully explored [5, 6]. Herein, we report the 1^st^ case of GV managed with this novel surgical approach.

## 2. Case

**Figure 1:**
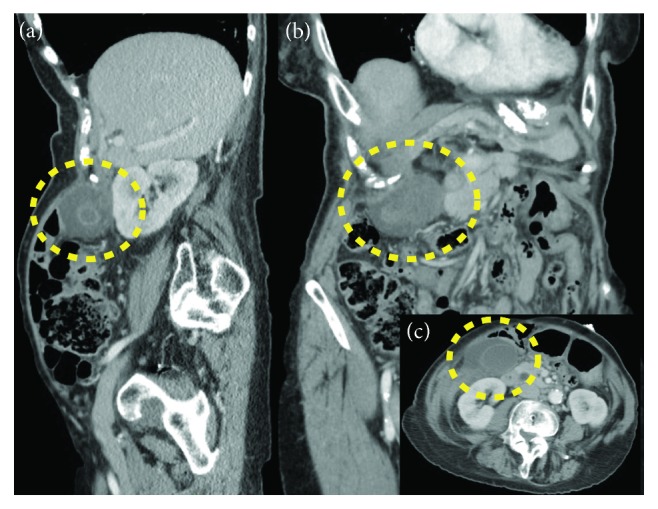
CT scan: (a) sagittal plane; (b) coronal plane; (c) axial plane. Dotted line circle shows the site of the gallbladder. Unique lithiasis is observed in (a) and (c).

**Figure 2:**
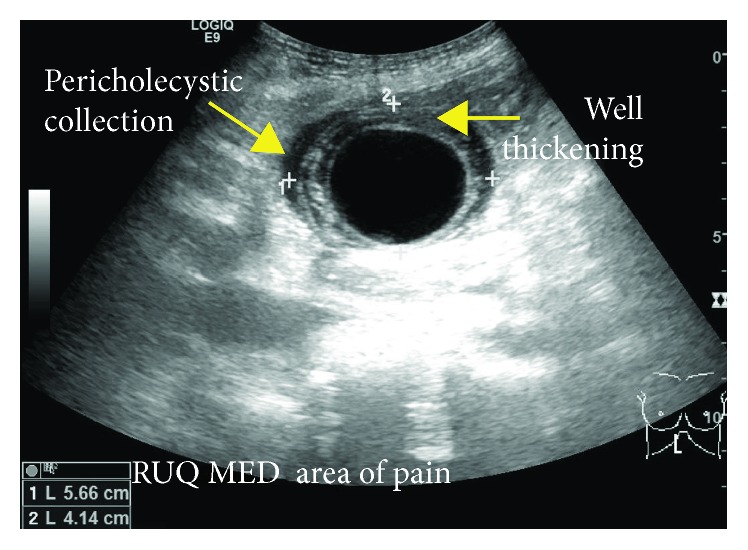
US scan.

This 81-year-old female presented to the emergency room with an acute onset of nonradiating and sharp right upper quadrant (RUQ) pain, primed by food intake and associated with nausea and fever (38.8°C). Past medical history was significant for hypertension, allergic sinusitis, and multiple abdominal surgeries (partial hysterectomy, right salpingo-oophorectomy, and open adhesiolysis for small bowel obstruction). Labs were unremarkable; WBC and liver function parameters were within normal limits. Abdominal and pelvic CT scans showed a dilated GB with large (5.4 cm) stone without signs of cholecystitis (Figure 1). RUQ ultrasound (US) reported a GB with marked wall thickening, pericholecystic fluid, and positive sonographic Murphy's sign (Figure 2). The patient was admitted from the emergency department to medical service to receive treatment for acute cholecystitis with IV antibiotics and pain management.

Shortly thereafter, the patient developed atrial fibrillation with rapid ventricular response, which required consultation and treatment by the cardiology consultant.

Surgical consultation was then requested, and the patient was brought to the operating room for an urgent robotic-assisted cholecystectomy.

Under general anesthesia, the patient was positioned supine, with tucked arms and intermittent compression devices in lower extremities. Pneumoperitoneum was achieved with a Veress needle placed at Palmer's point [7].

A 5 mm skin incision was performed over the right flank to place the laparoscopic camera. The initial diagnostic laparoscopy showed numerous omental and intestinal adhesions to the abdominal wall. Two additional 5 mm trocars were placed cephalad and caudal to the prior. Successful and uneventful adhesiolysis was carried out. The robotic trocars were then placed according to standard configuration for robotic cholecystectomy [8]. The patient was then placed in reverse Trendelenburg, and the Da Vinci Xi® cart was docked in from the patient's right side.

**Figure 3:**
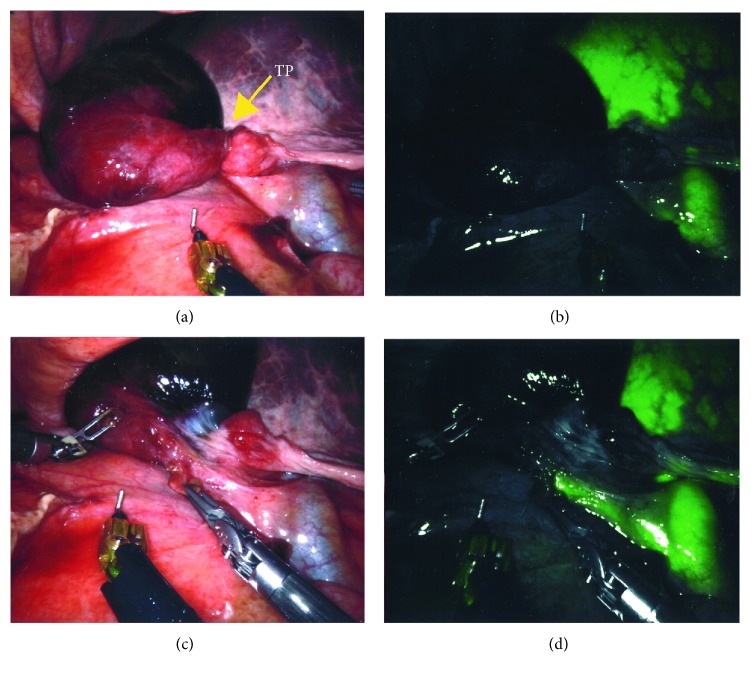
Comparison of bright vs. near-infrared images: (a) twisted GB in bright light view; (b) twisted GB in near-infrared view; (c) untwisted GB in bright light view; (d) untwisted GB in near-infrared view. TP: torsion point.

After the robotic arms were connected, further adhesiolysis was required in order to visualize the GB. The torsion of the GB hilum was identified, accompanied with a distended and gangrenous GB (Figure 3). The GB was attached to the liver bed over a small area in close proximity to the medial aspect of the fundus. The fundus was grasped with the 3^rd^ arm; the organ was untwisted (Figure 3) and then pulled cranially towards the diaphragm in order to expose the GB neck and also lift up the liver.

**Figure 4:**
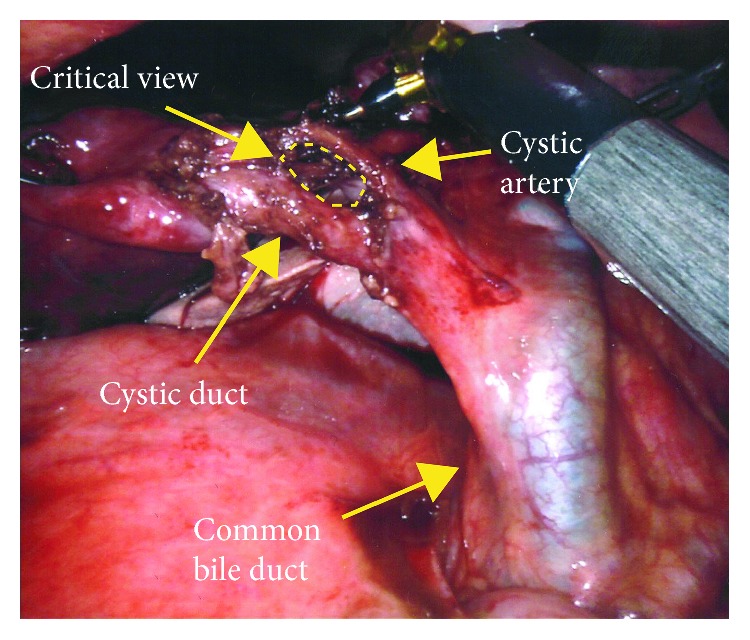
Hilar element dissection.

**Figure 5:**
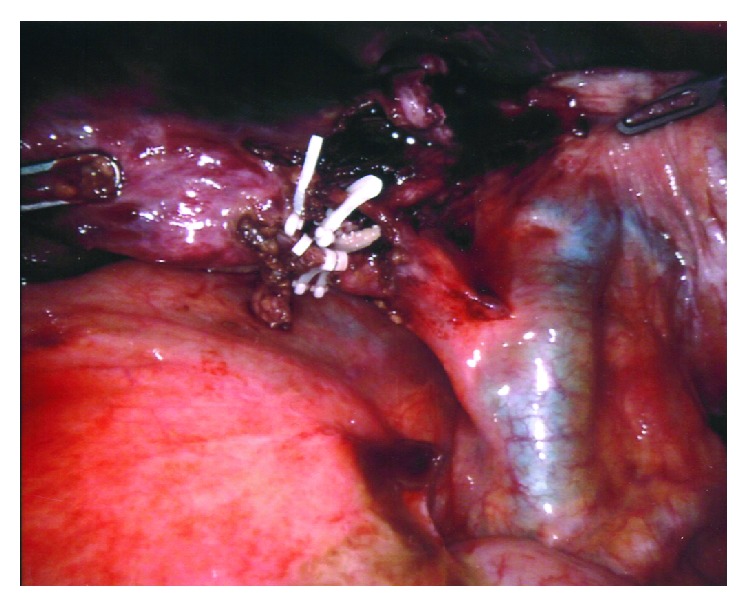
Clipped hilar elements before transection.

A Cadiere forceps and the robotic hook were used to dissect the hilum of the GB. After the cystic duct was identified, intravenous indocyanine green fluorescence (ICG) was used to facilitate the assessment of the biliary anatomy as shown in Figure 3 (IV ICG was previously injected at induction of general anesthesia). The cystic artery was then identified and dissected, and Calot's triangle was exposed adequately (Figure 4); the cystic duct and artery were transected in between Hem-o-loks with robotic scissors (Figure 5). The small attachment between the fundus and the liver bed was taken down with the cautery hook. Hemostasis was checked before the specimen was retrieved in an Endobag. The patient was transferred to the recovery room after surgery. Operative time was 126 min, and estimated blood loss was 25 ml.

The postoperatory course went uneventful; the patient was discharged on postoperatory day 2, with anticoagulation treatment for atrial fibrillation, ambulating, and with resolved pain. The pathology report informed gangrenous cholecystitis.

## 3. Discussion

GV is a rare condition that occurs after the GB twists around its mesentery [9]. It most frequently affects the elderly (seventies and eighties) and more commonly women at a ratio of 3 : 1 compared to men [9]. The main postulated contributing factors are loss of visceral fat, liver atrophy, and long mesentery [2]. These conditions result in a floating GB, predisposed to torsion [2]. Other precipitating factors that have been hypothesized are “violent” peristalsis of the neighboring organs, kyphosis, and atherosclerosis of the cystic artery [10]. The role of gallstones seems to be less relevant, because more than half of the patients with GV does not have gallstones [2].

According to the classification by Gross [11], free floating GBs can be classified into two groups: type A, when the mesentery supports the GB and the cystic duct, and type B, in which the mesentery only supports the cystic duct. In this case, the floating GB adopted the type A configuration.

Clinical symptoms are nonspecific, usually mimicking an acute cholecystitis (e.g., abdominal pain, nausea and vomiting, and palpable mass in RUQ). This is the reason why a high level of suspicion is a key. The low frequency of fever and jaundice, associated with a poor response to antibiotic therapy, may contribute to the differential diagnosis from acute cholecystitis [3]. If surgical intervention is delayed, due to GB necrosis and perforation, mortality associated due to GV is 6% [9]. The triad described by Lau et al. [12] may help to identify the patients who are more likely to present GV: (1) appearance (elderly, thin, and spinal deformities), (2) symptoms (sudden onset, early emesis, and RUQ pain), and (3) examination (nontoxic presentation, palpable abdominal mass, and pulse-temperature discrepancy). Laboratory results are usually nonspecific: while liver function tests are commonly under normal limits, elevated WBC is a frequent finding [10]. This stand in contrast to our case, in which WBC was under the normal limits.

Preoperative diagnosis of GV can be challenging, and most images may be interpreted as acute cholecystitis, especially if lithiasis is present [13]. Ultrasonography may describe a large and freely mobile GB, with a markedly thickened and multilayered wall [3]. In our case, acute cholecystitis signs were found, but freely mobile GB was not described. Naganuma et al. [14] proposed that color Doppler US may contribute to the differential diagnosis, because blood flow to the GB is interrupted in the setting of GV, whereas the flow of the cystic artery along the wall can be observed in acute cholecystitis. CT scan findings related to GV are distended GB, abrupt angulation of Hartmann's pouch, and change of the anatomical position of the GB from vertical to horizontal [13]. MRI may show high signal intensity within the GB wall on T1 signal (finding consistent with necrosis and hemorrhage) [10]. Finally, use of hydroxy-iminodiacetic acid (HIDA) scan was described in literature. A “bulls-eye” configuration from the accumulation of radioactivity in the GB can be observed in this case [15].

Surgical management must not be delayed if diagnosis is made preoperatively. A minimally invasive approach should be the first choice [10]. Despite being a relatively easy surgery (considering that almost no detachment from the liver bed is required to perform the cholecystectomy), attention should be paid to the proper identification of the structures of the hilum. Calot's triangle may be distorted due to the abnormal position of the structures [16], so it is recommended to assess the anatomy with diagnostic imaging. Intraoperatory cholangiogram is advised for the laparoscopic approach in order to avoid an unwanted biliary injury [17], but with the robotic approach, this is not necessarily required due to the integration of ICG fluorescence into the platform [6]. In our case, obtained near-infrared images with the aforementioned technique allowed to clearly identify the biliary structures, as seen in Figure 3.

Robotic surgery is on the rise. This novel technology is now used not only for routine but also for complex procedures [18, 19]. Despite the growth and expansion of applications, the use of the robotic platform in acute care surgery is still not fully explored [6]. Our case report presents a rare condition of the GB that has been successfully managed with the robotic approach in the acute setting [8]. Nevertheless, the laparoscopic approach, with widespread availability, lower costs, and proven safety [20], is still a valid approach for this pathology.

The magnified 3D vision, the finer dissection facilitated by instruments with Endowrist® (articulation in the distal part of the instrument that allows reproducing the freedom of movement of the human wrist), and the real-time assessment of biliary anatomy using ICG-aided cholangiography were perceived by the surgical team as added benefits to the more traditional laparoscopic approach. However, further evidence is necessary to show an obvious advantage of the robotic approach in the management of this rare and acute condition in comparison to the widely accepted laparoscopy.
